# Supplementary material for: Patterns of Intron Gain and Loss in Fungi
Source: PLoS Biol. 2004 Nov 30;2(12):e422. doi: 10.1371/journal.pbio.0020422 (PMC532390; doi:10.1371/journal.pbio.0020422)
Supplement: Table S1 — Also available at http://genes.mit.edu/NielsenEtAl/. (4.3 MB ZIP). [file pbio.0020422.st001.zip › NielsenEtAl/html/506.html]

AN7629.1.NCU09002.1.MG01667.1.FG05564.1


```
 CLUSTAL W (1.82) Multiple Sequence Alignments - Introns Inserted


Sequence 1: AN7629.1	84 aa
Sequence 2: MG01667.1	92 aa
Sequence 3: FG05564.1	92 aa
Sequence 4: NCU09002.1	93 aa
Alignment Length: 102 aa
Number Identitical Residues: 28 aa
Alignment Score (without introns) 1877


MG01667.1 	MFPSR2ALRAYQPGHSKPNITGFDPKTFVQAATKK----RYDPWERL2ESWRYTGQFSRF
NCU09002.1	MRVTR2VLRAVPK-----NPTGFNISEFRRAARVP----EHDPWIKN2ESWRTTGQFSRS
FG05564.1 	MLATR2VLRAAAGG-PKPNITGFNMRAFQASTGQP----RYDPWERN2EAWRYQGPYTRW
AN7629.1  	-----~-----MP---APNPTGFDINEFKAAANPRSVWAKRDPWARH2EAWRYTGPFSRF
          	                 .* ***:   *  ::   :  :. *** :  *:**  * ::* 

MG01667.1 	NRFKGAFPGLGIATVAFAAYCTYEHFFLKEDHHV-EGHH-----
NCU09002.1	ERFKGALPGFGTASVAFAIYCVYEHLFLKDKHHHDGTASAVTAH
FG05564.1 	NRLKSGFPGLGIATVAFTAYCGYEYFFLEDEHHHGEEHH-----
AN7629.1  	NRFRNIFPGFGIATVAFAGYCAYEHFFIKDDHHHGDSHH-----
          	:*::. :**:* *:***: ** **::*:::.** .
```
